# An antimicrobial stewardship program improves antimicrobial treatment by culture site and the quality of antimicrobial prescribing in critically ill patients

**DOI:** 10.1186/cc11854

**Published:** 2012-11-05

**Authors:** Christina M Katsios, Lisa Burry, Sandra Nelson, Tanaz Jivraj, Stephen E Lapinsky, Randy S Wax, Michael Christian, Sangeeta Mehta, Chaim M Bell, Andrew M Morris

**Affiliations:** 1 Department of Medicine, McMaster University, 1280 Main Street West, Hamilton, Ontario, L8S 4K1, Canada; 2 Department of Pharmacy, Mount Sinai Hospital, 600 University Avenue, Toronto, Ontario, M5G 1X5, Canada; 3 Office of Quality and Performance Measurement, Mount Sinai Hospital, 600 University Avenue, Toronto, Ontario, M5G 1X5, Canada; 4 Department of Medicine, University of Toronto, 200 Elizabeth Street, Toronto, Ontario, M5G 2C4, Canada; 5 Department of Medicine, Queen's University and University of Toronto, Lakeridge Health, 1 Hospital Court, Oshawa, Ontario, L1G 2B9, Canada

## Abstract

**Introduction:**

Increasing antimicrobial costs, reduced development of novel antimicrobials, and growing antimicrobial resistance necessitate judicious use of available agents. Antimicrobial stewardship programs (ASPs) may improve antimicrobial use in intensive care units (ICUs). Our objective was to determine whether the introduction of an ASP in an ICU altered the decision to treat cultures from sterile sites compared with nonsterile sites (which may represent colonization or contamination). We also sought to determine whether ASP education improved documentation of antimicrobial use, including an explicit statement of antimicrobial regimen, indication, duration, and de-escalation.

**Methods:**

We retrospectively analyzed consecutive patients with positive bacterial cultures admitted to a 16-bed medical-surgical ICU over 2-month periods before and after ASP introduction (April through May 2008 and 2009, respectively). We evaluated the antimicrobial treatment of positive sterile- versus nonsterile-site cultures, specified *a priori*. We reviewed patient charts for clinician documentation of three specific details regarding antimicrobials: an explicit statement of antimicrobial regimen/indication, duration, and de-escalation. We also analyzed cost and defined daily doses (DDDs) (a World Health Organization (WHO) standardized metric of use) before and after ASP.

**Results:**

Patient demographic data between the pre-ASP (*n *= 139) and post-ASP (*n *= 130) periods were similar. No difference was found in the percentage of positive cultures from sterile sites between the pre-ASP period and post-ASP period (44.9% versus 40.2%; *P *= 0.401). A significant increase was noted in the treatment of sterile-site cultures after ASP (64% versus 83%; *P *= 0.01) and a reduction in the treatment of nonsterile-site cultures (71% versus 46%; *P *= 0.0002). These differences were statistically significant when treatment decisions were analyzed both at an individual patient level and at an individual culture level. Increased explicit antimicrobial regimen documentation was observed after ASP (26% versus 71%; *P *< 0.0001). Also observed were increases in formally documented stop dates (53% versus 71%; *P *< 0.0001), regimen de-escalation (15% versus 23%; *P *= 0.026), and an overall reduction in cost and mean DDDs after ASP implementation.

**Conclusions:**

Introduction of an ASP in the ICU was associated with improved microbiologically targeted therapy based on sterile or nonsterile cultures and improved documentation of antimicrobial use in the medical record.

## Introduction

Antimicrobial use in the intensive care unit (ICU) is seemingly ubiquitous. An international, prospective, point prevalence study of more than 1,200 ICUs documented that 71% of ICU patients received antimicrobials [1]. This widespread use may be inappropriate, with recent studies estimating as many as 30% of regimens are unnecessary [2-5]. The consequences of unnecessary antimicrobial use (antimicrobial resistance, adverse events, and cost) necessitate judicious use.

Antimicrobial stewardship programs (ASPs) represent organizational approaches to harmonize competing concerns of adequate antimicrobial coverage, adverse events, and resistance, and are well suited to ICU settings [4,6]. A position statement from the Infectious Disease Society of America (IDSA), the Society for Healthcare Epidemiology of America (SHEA), and the Pediatric Infectious Disease Society (PIDS), deemed stewardship a "fiduciary responsibility for all healthcare institutions" and recommended mandatory implementation [7].

In critically ill patients, one study documents up to 50% of positive cultures may actually represent contamination [8]. Positive microbial cultures in critically ill patients often prompt reflexive antimicrobial therapy, regardless of the sampling site or the contamination potential [8]. Specifically, positive "sterile site" cultures (such as blood cultures) better represent true infection than do positive "nonsterile site" cultures (such as wound cultures). Nonsterile sites are more likely to reflect colonization or contamination. Appropriate antimicrobial use should preferentially treat positive cultures from sterile sites rather than from nonsterile sites. Distinguishing between contamination and true positives can be difficult [8], and clinicians may benefit from ASP assistance with regimen choice and education surrounding contamination potential.

Although the literature suggests that ASPs are associated with reduced ICU antimicrobial utilization [7], how these results are achieved on an individual patient level is unclear. Our objective was to determine whether the introduction of an ASP in a medical-surgical ICU altered the decision to treat positive sterile versus nonsterile culture sites. The IDSA/SHEA/PIDS consensus statement [7] recommends "address[ing] deficiencies in education" to improve antimicrobial practice. We developed an intervention of academic detailing by our ASP during medical-surgical ICU rounds. We hypothesized that this intervention would alter provider decisions to treat positive sterile versus nonsterile culture sites and that an improved understanding would be manifest in higher quality documentation of antibiotic decisions.

A previous study examined videotaped recordings of resident/patient encounters and medical documentation [9]. This study demonstrated that residents in their second year produced better documentation than did first-year residents. This study inferred that knowledge and experiential teaching play a role in chart-documentation practices [9]. Another study by Carroll *et al. *[10] demonstrates that daily progress notes written by resident physicians in the neonatal ICU often contain inaccurate, or omit, pertinent information. Documentation inaccuracy or omission usually surrounds items that residents feel uncomfortable managing; such as vascular lines and medications. We postulate that our educational ASP may help to chart better documentation practices by discussing antimicrobial status with an infectious disease physician and/or pharmacist.

Inappropriate overuse results from inappropriate assessment of risk, incorrect utilization and/or interpretation of tests suggesting infection (for example, white blood cell count), and prolonged duration of therapy. By suggesting to physicians, for example, that isolated leukocytosis is not an indication for "panculturing" or that wound swabs are of limited value in diagnosing infection, the ASP influences practice.

## Materials and methods

### Overview

A quasi-experimental uncontrolled intervention was used to determine the impact of an ASP on the treatment of positive sterile-site compared with nonsterile-site cultures. The institution's Research Ethics Board approved this study and waived the need for informed consent, given the retrospective design.

### Patients

We reviewed consecutive patients older than 18 years, who were admitted to the ICU, had positive microbial cultures, and received antimicrobials. The first group of patients was admitted to the ICU during April and May 2008 and represented the pre-ASP period, and a second group of patients admitted during April and May 2009 represented ICU practices after ASP introduction. The 2-month period was used as a convenience sample. The ASP was introduced to the institution in February 2009, and we selected April/May 2008 and 2009 for data collection to allow maturation of the intervention as well as to control temporal and seasonal effects.

### Setting

Mount Sinai Hospital is a 472-bed teaching hospital affiliated with the University of Toronto. The medical-surgical ICU is a closed unit, with 16 active beds and staff intensivists, fellows, residents, a dedicated ICU pharmacist, and other allied health personnel. The ICU occupancy in 2008 and 2009 was 82% and 80%, respectively. Approximately 13% of bed occupancy involves patients with hematologic malignancies.

### Intervention

Our ASP approach centered on education, expert consultation, prospective audit, and feedback [11]. The ASP team, which included a dedicated ASP pharmacist and an infectious disease consultant, interacted with prescribers in a collaborative and educational capacity. The ASP aimed to curtail unnecessary use by engaging the prescriber in frequent, scheduled, face-to-face clinical-decision support, in the setting of minimal electronic decision-support tool availability. Each weekday, the ASP team met with the ICU team after routine ICU rounds, and reviewed each ICU patient. Recommendations for individual patients were based on clinical, radiographic, and laboratory data, including microbiology results. Recommendations were made for further investigations, antimicrobial agents, de-escalation, and the need for formal infectious-disease consultation (a routine consultation service supervised by an attending infectious-disease staff physician). The ICU team was free to consult the infectious-disease service as they saw fit, and compliance to the infectious-disease team was not recorded. Similarly, adherence to the ASP team recommendations (which is separate from the infectious-disease team, although also led by an infectious-disease physician) was optional for the ICU team. The face-to-face interaction between ASP and ICU team took approximately 1 minute per patient per day.

### Data collection

Data were collected retrospectively from the patient's paper and electronic charts. Parameters collected included demographic data, culture results, antimicrobial regimen, ICU chart documentation practices, cost, and defined daily doses (DDDs) per 1,000 patient-days. Culture results were recorded and stratified into sterile and nonsterile sites, specified *a priori*. Sterile sites included blood cultures, bronchoalveolar lavage (BAL), pleural fluid, cerebrospinal fluid, peritoneal fluid, bile, pancreatic fluid, and muscle tissue. Nonsterile sites included sputum, nasopharyngeal swabs, urine, wound, nail, skin, stool, genital swabs, rectal swabs, and gastric aspirates.

Patient charts were reviewed by one reviewer, CK, for standardization. The reviewer was blinded during data abstraction. The daily ICU progress notes were analyzed exclusively for details surrounding documentation. The daily ICU notes are written each day for the patients' length of stay in the ICU. A binary yes/no decision for "appropriate documentation" was made for each daily progress note independently. The percentage of appropriate documentation was calculated with the number of appropriately documented daily ICU notes divided by the total daily ICU notes. The daily progress notes were chosen in lieu of the nursing Kardex, nursing notes, pharmacist notes, or daily prescription as a measure of medical-learner behavioral change, one of the cited areas of deficiency in the recent IDSA/SHEA/PIDS consensus statement on ASPs [7]. Charts were reviewed for documentation of three specific details: (a) regimen and indication, (b) duration, and (c) de-escalation. These parameters were collected as binary "yes/no" data points ("yes" deemed optimal documentation, and "no" deemed inappropriate documentation or the absence of documentation).

Documentation of regimen/indication was defined as the documentation of antimicrobial agent by name and indication (for example, ceftriaxone, 2 g IV, for suspected meningitis). The abbreviated documentation of "on antibiotics" was deemed inappropriate documentation. Charts were deemed to have "no documentation" when the antimicrobial agent was not overtly documented, infectious-disease section of daily note omitted, or when the indication was not explicit.

Antimicrobial duration was defined as explicit start and stop dates for antimicrobial regimens, or specific duration of therapy (for example, 3 days). Documentation was deemed adequate if the criteria for appropriate regimen/indication were also met. The abbreviated documentation of "2-week course" without an explicitly named antimicrobial agent or indication was deemed inappropriate documentation. We chose to define this parameter in this manner as a teaching point for junior learners; for example, if the learner consistently documents "trimethoprim/sulfamethoxazole, 160 mg PO BID, for 3 days for uncomplicated urinary tract infection," the learner is more likely to learn and remember duration and indication. The percentage of appropriate documentation was calculated by the number of daily ICU notes fulfilling the definition of appropriate regimen/indication documentation and appropriate duration documentation divided by the number fulfilling the definition of appropriate regimen/indication.

De-escalation of antimicrobial therapy was defined as parenteral-to-enteral route exchange, agent exchange to tailor spectrum of activity (based on culture results), and discontinuation of unnecessary agents, as per prior definition in the literature [5]. Documentation was deemed adequate if the criteria for appropriate regimen/indication were met in addition to de-escalation details. The abbreviated documentation of "antibiotics tailored," "antibiotics de-escalated," or "cultures specified, antibiotics changed" without any other specifics were deemed inappropriate documentation. The percentage of appropriate documentation was calculated by the number of daily ICU notes fulfilling appropriate regimen/indication definition and de-escalation definition divided by the number fulfilling the regimen/indication definition.

The ASP team did not contribute to medical records, and thus, did not contribute to better chart documentation practices.

Decisions regarding treatment of positive nonsterile cultures and a clinical picture consistent with an infection in that organ system was at the discretion of the treating ICU physician and is a tenet of ASP rounds. The ASP team may suggest that the culture represents contamination, but if the clinical picture is one of infection, the ICU team may disregard ASP suggestions, thereby preserving ICU prescribing autonomy.

Our study did not address the issue of prescribing antimicrobials before cultures are drawn as the Surviving Sepsis guidelines [12] advocate for the initiation of broad-spectrum antibiotics followed by appropriate de-escalation. We sought to examine the ASP influence on ICU prescribing practices based on culture site only after the final culture report was returned. For example, empiric antimicrobials that were continued after positive nonsterile culture results returned were deemed "treated nonsterile culture sites." Likewise, empiric antimicrobials that were stopped when culture results returned were counted as "nontreated."

We sought to examine use of antimicrobials before and after ASP, and used DDD as our metric. DDDs are defined by the World Health Organization (WHO) as the assumed average maintenance dose per day for a medication used for its main indication in adults [13]. The DDD is assigned by the WHO for medications that have a WHO Anatomical Therapeutic Chemical code. The DDD is a unit of measure that may not necessarily reflect the recommended or prescribed daily dose. DDDs provide estimates of drug consumption independent of cost [13]. We use the DDD to assess the trends in drug consumption and to perform comparisons between groups, as recommended by the WHO [13]. DDDs were calculated monthly based on dispensing data from the pharmacy system (PharmaNet Inpatient, Cerner Corporation, Kansas City, MO, USA). To calculate DDDs, the dispensed antimicrobial quantity (in grams) was divided by the WHO DDD. This result was then divided by the ICU patient days and then multiplied by 1,000 to obtain DDDs/1,000 patient days as a standardized metric. Costs of antimicrobials dispensed were obtained from reports by using the Pharmacy Department's PharmaNet pharmacy software (PharmaNet Development Group, Princeton, NJ, USA) and are reported in Canadian dollars. All costs over the 2-month period of April and May 2008 and 2009, respectively, were summed to give a total cost of antimicrobials. Antimicrobial costs were also calculated per patient-day in the ICU. Costs and DDD data will be presented as descriptive statistics, as they are not a primary outcome of this study. Costs of antimicrobials were not standardized; however, they did not change significantly within the 2-year period of the study.

### Statistical analysis

Regimens by sample-site sterility and ICU documentation practices were analyzed by using the χ^2 ^test, with one degree of freedom and Yates continuity correction. Descriptive statistics such as absolute percentage decreases or increases over the study time period were used to analyze cost and DDDs. As a measure of quality improvement, we calculated a novel measure, the Sample Sterility Ratio (SSR, which is the ratio of sterile-site samples treated to nonsterile-site samples treated), and followed this metric to document treatment effect over time. We calculated a percentage for chart documentation in each of the three parameters (antimicrobial regimen/indication, duration, and de-escalation). Microsoft Excel (Microsoft Corp., Redmond, WA, USA) was used for analysis. A *P *< 0.05 was considered significant.

## Results

In total, 269 patients were admitted to the medical-surgical ICU during the 2-month pre-ASP period (*n *= 139) and the 2-month post-ASP period (*n *= 130) requiring antimicrobials. Demographics of the pre-ASP and post-ASP groups were similar (Table 1). A statistically significant increase was found in the number of formal infectious disease consultations after ASP (nine versus 22; *P *= 0.013).

**Table 1:** Demographic and baseline characteristics of the patients

	Before ASP *(n *= 139)	After ASP *(n *= 130)	*P *value
Male	62 (45%)	67 (52%)	0.310
Older than 70 years	50 (36%)	42 (32%)	0.614
Medical admission	89 (64%)	90 (69%)	0.439
Transferred from external institution	44 (32%)	52 (40%)	0.194
APACHE II (mean ± SD)	21.9 ± 6.2	22.6 ± 5.2	0.318
Malignancy	42 (30%)	30 (23%)	0.237
Previous HSCT	2 (1%)	8 (6%)	0.085
Renal replacement therapy	13 (9%)	13 (10%)	0.858
Infectious Disease Service consultations	9 (7%)	22 (17%)	0.013
ICU mortality	26 (19%)	18 (13%)	0.362

Malignancy, active malignancy (cancer within in past 6 months, or currently receiving treatment such as radiation or chemotherapy) or malignancy within 10 years before admission; HSCT, hematopoietic stem cell transplant (defined as autologous or allogenic stem cell transplantation within the past 5 years and includes both myeloablative and nonmyeloablative transplant protocols); renal replacement therapy, all forms of dialysis (intermittent hemodialysis, continuous renal replacement therapy, and so on).

Before ASP, 225 positive cultures were used versus 179 after ASP. The number of sterile cultures was similar between the pre-ASP period and post-ASP period (101 versus 72; *P *= 0.401) (Table 2). The number of cultures by culture type was similar between the pre- and post-ASP periods, except for a significant increase in the number of positive sputum cultures in the post-ASP period (38 versus 48; *P *= 0.036)

**Table 2:** Distribution of positive sterile and nonsterile cultures

	Before ASP	After ASP	*P *value
Sterile cultures	101	72	
Blood	70 (69%)	55 (76%)	0.394
Other^a^	31 (31%)	17 (24%)	0.394
Nonsterile cultures	124	107	
Sputum	38 (31%)	48 (45%)	0.036
Urine	44 (35%)	36 (34%)	0.877
Wound	25 (20%)	14 (13%)	0.209
Other^b^	17 (14%)	9 (8%)	0.288

^a^Includes pleural fluid, pancreatic fluid, bronchoalveolar lavage, and muscle tissue. ^b^Includes arterial and venous catheter tips, skin, and rectal cultures.

A higher proportion of sterile-site cultures was treated after ASP (64% versus 83%; *P *= 0.01), and a lower proportion of nonsterile-site cultures was treated (71% versus 46%; *P *= 0.0002). The majority of untreated sterile cultures were coagulase-negative staphylococci in blood cultures or positive BAL cultures, likely representing contaminants.

We repeated the analysis as a per-patient-based analysis as well as a per-culture-based analysis. This was done because we identified patients who had only positive sterile-site cultures, others who had only positive nonsterile-site cultures, and a group that had both positive sterile-site and positive nonsterile-site cultures. In the per-patient-based analysis, a higher proportion of patients with only positive sterile-site cultures were treated after ASP (70% versus 91%; *P *= 0.0082) and a lower proportion of patients with only nonsterile-site cultures were treated after ASP (94% versus 48%; *P *= 0.0001). No statistical difference was found in treating patients who had both positive sterile- and nonsterile-site cultures after ASP (67% versus 42%; *P *= 0.3632) (Table 3).

**Table 3:** Treatment of sterile versus nonsterile sites: per patient analysis

	Before ASP	After ASP	*P *value
Patients	139	130	
Patients with only positive sterile cultures	71	56	
Patients treated	50 (70%)	51 (91%)	0.0082
Patients with both positive sterile and positive nonsterile cultures	15	12	
Patients treated	10 (67%)	5 (42%)	0.3632
Patients with only positive nonsterile cultures	53	62	
Patients treated	50 (94%)	30 (48%)	0.0001

Statistically significant results were also found when the analysis was repeated as a per-culture-based analysis. A higher proportion of the cultures from patients with only positive sterile-site cultures were treated after ASP (61% versus 90%; *P *= 0.0002) and a lower proportion of the cultures from patients with only nonsterile-site cultures were treated after ASP (89% versus 47%; *P *= 0.0001). No statistical difference was found in treating cultures from patients who had both positive sterile- and nonsterile-site cultures after ASP (52% versus 44%; *P *= 0.6139; Table 4).

**Table 4:** Treatment of sterile versus nonsterile sites: per culture analysis

	Before ASP	After ASP	*P *value
Positive cultures	225	179	
Sterile	101 (45%)	72 (40%)	0.401
Nonsterile	124 (55%)	107 (60%)	0.401
Sterile cultures treated	65 (64%)	60 (83%)	0.01
Nonsterile cultures treated	88 (71%)	49 (46%)	0.0002
Number of cultures of patients with only positive sterile cultures	86	60	
Cultures treated	52 (61%)	54 (90%)	0.0002
Number of cultures of patients with both positive sterile and positive nonsterile cultures	60	32	
Cultures treated	31 (52%)	14 (44%)	0.6139
Number of cultures of patients with only positive nonsterile cultures	79	87	
Cultures treated	70 (89%)	41 (47%)	0.0001

Increasingly detailed ICU chart documentation was available after ASP. A higher proportion of charts detailed antimicrobial regimen/indication after ASP (26% versus 71%; *P *< 0.0001). After ASP, an 18% absolute increase (53% versus 71%; *P *< 0.0001) was found in the number of regimens with formally documented stop dates and a significant increase in de-escalation of antimicrobials (15% versus 23%; *P *= 0.026).

**Figure 1:**
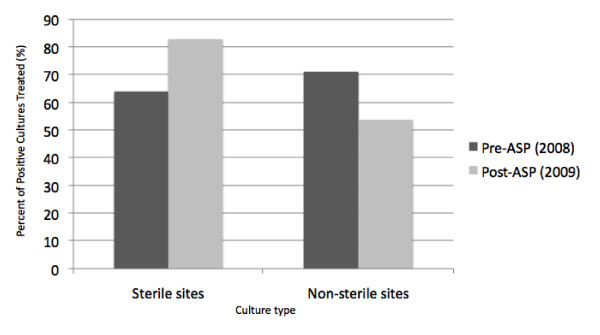
Treated cultures before and after ASP implementation separated by culture designation.

The Sample Sterility Ratio increased from 0.9 before ASP to 1.54 after ASP. Treated cultures before and after ASP implementation are graphically detailed in Figure 1, separated by culture designation.

ICU antimicrobial costs decreased in the post-ASP period ($60,610 before ASP versus $43,857 after ASP) (Table 5). No appreciable changes in drug acquisition costs were found for the hospital over the study period. Antimicrobial use was 13.7% higher in the pre-ASP period compared with the post-ASP period (3,592 versus 3,010 DDD/1,000 patient days) (Table 5).

**Table 5:** Antimicrobial use, cost, and chart documentation

	Before ASP	After ASP	Change {%}
DDD/1,000 patient days	3,592	3,100	-13.7
Antimicrobial costs	$60,610	$43,866	-27
Antimicrobial costs/patient day	$71	$54	-24

			***P *value**

Antimicrobial regimen and indication documentation	206/779 (26%)	467/662 (71%)	<0.0001
Antimicrobial duration	109/206 (53%)	336/467 (71%)	<0.0001
Antimicrobial de-escalation	31/206 (15%)	107/467 (23%)	0.026

DDD, Defined daily doses; costs, in Canadian dollars.

## Discussion

After the introduction of ASP, we observed a statistically significant increase in the treatment of sterile-site cultures and a reduction in the treatment of nonsterile-site cultures, in both our per-patient and per-culture-based analyses. Increasingly detailed chart documentation was observed after ASP, with an increase in formally documented stop dates, as well as reductions in antimicrobial use and costs after ASP implementation. We calculated a novel measure, the Sample Sterility Ratio, which was increased after ASP.

Multiple studies report benefits of ASP implementation, demonstrated in both hospital patients and critically ill patients [4,6,7]. No literature reports detail treatment choice by culture site, or ICU chart documentation after ASP. To our knowledge, this is the only study to document the change in processes of care that accompany the introduction of an ASP in the ICU: improved documentation of antimicrobial therapy and more judicious attention to the quality and sterility of positive microbiologic samples. Only one other study in the literature examines chart documentation in the ICU [14]. This prospective before and after 7-month intervention study in a medical ICU in France documented similar findings to those of our study, increased reassessment of antimicrobial prescriptions after the intervention [14].

A few studies detail educational relations between the ASP and ICU teams. Our results may have considerable merit to hospital administrators, who may consider ASP implementation as a cohesive approach of instituting better antimicrobial practices. The IDSA/SHEA/PIDS policy statement warns against the "rapidly dwindling antimicrobial armamentarium" and names ASPs "fiduciary responsibilities for all healthcare institutions" [7]. They recommend the "creation of a multidisciplinary interprofessional team" similar in structure to our intervention [7]. The guidelines state that

significant knowledge deficits in the areas of antimicrobial stewardship are prevalent....and that educational programs should be developed...because of the gravity of the problems with antimicrobial resistance that confront society and the paucity of readily available clinical solutions [7].

Strengths of our study include the attention to prescriber education in the ASP structure. Education is widely considered the foundation of a successful ASP [7,9] yet a paucity of dedicated evidence supports this assertion. The optimal constituents of a mutually beneficial educational ASP program are unknown. Our ASP educates prescribers directly at the point of care, as well as through audit and feedback. Our strategy facilitates change on a case-by-case basis, and we here demonstrate that this strategy produces measurable change in both process and outcomes related to antimicrobial prescribing (antimicrobial use by culture site, chart documentation, cost, and use).

Another strength of our study is the statistically significant findings of increased treatment of sterile-site cultures and decreased treatment of nonsterile-site cultures in both a per-patient and a per-culture analysis. We believe the consistent statistical significance in both analyses demonstrates the rigor of our methods.

Another strength of our study includes a quantification of better chart-documentation practices after ASP. A previous study demonstrated that antimicrobial indication was recorded in 64% of patients before feedback and in 86.5% after feedback (*P *= 0.004) [15]. Our findings indicate a similar change. This is important because senior consultation advice and pertinent clinical infectious signs are rarely recorded [15], making indication sometimes difficult to elucidate in the daily ICU note. Without transparent chart documentation, it becomes increasingly difficult for other clinicians to make informed decisions. In an academic ICU setting with multiple consultants and high turnover of relatively junior housestaff, better documentation improves communication and may increase patient safety.

Our study has limitations. It is a single-center, uncontrolled, quasi-experimental study. Our data may reflect local prescription bias, formulary contracts, patient demographics, and ICU culture unique to our center, which may limit generalizability. Our data may also reflect a unique ASP relationship with the ICU team, which may not be replicable. In addition, our 2-month data may not reflect long-term practice or demonstrate durability of effect. However, the study results are similar to those of other studies that demonstrate the multiple benefits of an institutional ASP [5-7].

Another limitation of our study may be the choice of counting daily progress notes for documentation purposes. A number of areas in the patient's chart may document antimicrobial use (for example, doctor's orders, sign-out sheet). However, counting each of these as single documentation events would have been difficult logistically, so we chose the daily progress notes (which are completed on ICU team rounds and represent the team's plan for the patient for that day) as a representation of documentation, noting that this may be incomplete.

Another limitation to our study may the classification of bronchoalveolar lavage (BAL) as a "sterile" site. BALs at our institution represent the "best" airway culture site, as nasopharyngeal swabs and endotracheal aspirates may have more-frequent contamination. We understand that BALs have their own risk of potential contamination; however, we note that the American Thoracic Society still endorses BAL diagnostic use for several respiratory infections.

Another limitation may be the use of DDD as our metric for pharmacologic outcomes. DDDs may underestimate renal antimicrobial dosing (relevant in centers with a high incidence of renal insufficiency) [16]. We have also introduced a new metric, the SSR. This metric may be calculated and serially followed to document treatment effect; however, it has not been prospectively validated.

Limited previous studies demonstrate measurable outcomes without formulary restriction [17]. Considerable attention is attributed to successful ASPs with formulary restriction or preauthorization [17], and some may criticize our ASP model for its lack of such methods. Formulary restriction consists of decreased accessibility to certain controlled antimicrobials. It is postulated that formulary restriction will not change fervent prescriber beliefs, and thus may not have consistent or sustained impact on prescription patterns [4]. Additionally, formulary restriction does not address: (a) the potential need for source control; (b) the use of antimicrobials when they are not indicated at all; (c) the occasional need for broader/specific coverage; and (d) appropriate duration of therapy. The most common consequence of formulary restriction is "squeezing the balloon" (that is, by limiting the use of one or several antimicrobial agents, other antimicrobial use is inflated) [18,19]. We also believe it is an onerous ASP intervention that requires additional personnel and is often more constabulary than collegial. It is also perceived to diminish prescribing autonomy [4,20]. Although such programs are shown to decrease antimicrobial use, once the approval process is withdrawn, use of the particular antimicrobial increases substantially [20]. We have shown that with an ASP without formulary restriction focused on education, formal review and face-to-face clinical decision support can produce measurable behavioral change. We believe this has a twofold benefit: in the short term, to help the discussed patient, and in the long term, benefit of education, which may change practice and prescriber culture. Previous audit and feedback strategies vary and have produced inconsistent results. We have tried to produce a more-effective feedback ASP intervention by incorporating previously successful strategies in the literature [21-23] with our own nonrestricted formulary based face-to-face decision support.

Ongoing evaluation is required to measure long-term cost and patient-outcome implications of our ASP. Studies of prescriber and learner behavioral changes may be carried out to ensure the longevity of better chart-documentation practices and retention of appropriate antimicrobial use.

## Conclusions

Appropriate and judicious antimicrobial use guided by an ASP can be associated with significant benefit in critically ill patients. ASPs guide the clinician, measure adherence and performance, and ultimately benefit patients. Our ASP team minimized unnecessary use by sterile and nonsterile culture site, improved chart documentation, and decreased antimicrobial costs and overall use. Our ASP may warrant consideration by other teaching centers as a way to provide opportunities for education and collaboration, to conserve prescriber autonomy, and ultimately to produce beneficial clinical outcomes and enhance patient safety.

## Key messages

• This type of antimicrobial stewardship model educates the prescriber directly at the point of care, as well as through audit and feedback.

• This type of antimicrobial stewardship model does not use formulary restriction and preserves prescriber autonomy.

• A statistically significant increase was noted of treating sterile-site cultures after ASP and treating fewer nonsterile-site cultures, both by per-culture and by per-patient analysis.

• Increasingly detailed chart documentation surrounding antimicrobial regimens was observed after ASP, which may enhance patient safety.

## Abbreviations

ASP: Antimicrobial Stewardship Program; BAL: bronchoalveolar lavage; DDD: defined daily dose; ICU: intensive care unit; IDSA: Infectious Disease Society of America; PIDS: Pediatric Infectious Disease Society; SHEA: Society of Healthcare Epidemiology of America; SSR: sample sterility ratio; WHO: World Health Organization.

## Competing interests

The authors declare that they have no competing interests.

## Authors' contributions

CK carried out the data acquisition, participated in the analysis, and drafted the manuscript. AMM conceived of the study, participated in its design and coordination, and helped to draft the manuscript. CB spent considerable time revising the manuscript. LB and SN coordinated the pharmacy data. TJ coordinated the mortality and cost data. LB, SN, TJ, SL, RW, MC, SM, CB, TS, and AM participated in the initiation of the ASP at Mount Sinai Hospital. All authors read and approved the final manuscript.

## References

[B1] Vincent JL Rello J Marshall J Silva E Anzueto A Martin CD Moreno R Lipman J Gomersall C Sakr Y Reinhart K for the EPIC-II group of investigators International study of the prevalence and outcomes of infection in intensive care units JAMA 2009 16 2323 2329 10.1001/jama.2009.1754 19952319

[B2] Gandhi TN DePestel DD Collins CD Nagel J Washer LL Managing antimicrobial resistance in intensive care units Crit Care Med 2010 16 S315 S323 20647789 10.1097/CCM.0b013e3181e6a2a4

[B3] Colardyn F Appropriate and timely empirical antimicrobial treatment of ICU infections: a role for carbapenems Acta Clin Belg 2005 16 51 62 16082989 10.1179/acb.2005.011

[B4] Morris AM Stewart TE Shandling M McIntaggart S Liles WC Establishing an Antimicrobial Stewardship Program Healthcare Q 2010 16 64 70 10.12927/hcq.2013.21672 20357548

[B5] Hecker MT Aron DC Patel NP Lehmann MK Donskey CJ Unnecessary use of antimicrobials in hospitalized patients: current patterns of misuse with an emphasis on the antianaerobic spectrum of activity Arch Intern Med 2003 16 972 978 10.1001/archinte.163.8.972 12719208

[B6] Kaki R Elligsen M Walker S Simor A Palmay L Daneman N Impact of antimicrobial stewardship in critical care; a systematic review J Antimicrob Chemother 2011 16 1223 1230 10.1093/jac/dkr137 21460369

[B7] Society for Healthcare Epidemiology of America, Infectious Disease Society of America, Pediatric Infectious Diseases Society Policy statement on antimicrobial stewardship by the Society for Healthcare Epidemiology of America (SHEA), the Infectious Disease Society of America (IDSA) and the Pediatric Infectious Diseases Society (PIDS Infect Control Hosp Epidemiol 2012 16 322 327 22418625 10.1086/665010

[B8] Shafazand S Weinacker AB Blood cultures in the critical care unit: improving utilization and yield Chest 2002 16 1727 1736 10.1378/chest.122.5.1727 12426278

[B9] Moran MT Wiser TH Nanda J Gross H Measuring medical residents' chart-documentation practices J Med Educ 1988 16 859 865 3184152 10.1097/00001888-198811000-00006

[B10] Carroll AE Tarczy-Hornoch P O'Reilly E Christakis DA Resident documentation discrepancies in a neonatal intensive care unit Pediatrics 2003 16 976 980 10.1542/peds.111.5.976 12728074

[B11] Dellit TH Owens RC McGowan JE Jr Gerding DN Weinstein RA Burke JP Huskins WC Paterson DL Fishman NO Carpenter CF Brennan PJ Billeter M Hooton TM Infectious Diseases Society of America; Society for Healthcare Epidemiology of America Infectious Disease Society of America and the Society for Healthcare Epidemiology of America guidelines for developing an institutional program to enhance antimicrobial stewardship Clin Infect Dis 2007 16 159 177 10.1086/510393 17173212

[B12] Dellinger RP Levy MM Carlet JM Bion J Parker MM Jaeschke R Reinhart K Angus DC Brun-Buisson C Beale R Calandra T Dhainaut JF Gerlach H Harvey M Marini JJ Marshall J Ranieri M Ramsay G Sevransky J Thompson BT Townsend S Vender JS Zimmerman JL Vincent JL for the International Surviving Sepsis Campaign Guidelines Committee Surviving Sepsis Campaign: International guidelines for management of severe sepsis and septic shock Crit Care Med 2008 16 296 327 10.1097/01.CCM.0000298158.12101.41 18158437

[B13] World Health Organization: Collaborating Center for Drug Statistic Methodology. ATC Index with DDDs http://www.whocc.no/atcddd/

[B14] 14. Pulcini C Dellamonica J Bernardin G Molinari N Sotto A Impact of an intervention designed to improve the documentation of the reassessment of antibiotic therapies in an intensive care unit Med Mal Infect 2011 16 546 552 10.1016/j.medmal.2011.07.003 21855239

[B15] Seaton RA Nathwani D Phillips G Millar R Davey P Clinical record keeping in patients receiving antibiotics in hospital Health Bull (Edinb) 1999 16 128 133 12811903

[B16] Polk RE Fox C Mahoney A Letcavage J MacDougall C Measurement of adult antibacterial drug use in 130 US hospitals: comparison of defined daily dose and days of therapy Clin Infect Dis 2007 16 664 670 10.1086/511640 17278056

[B17] Fishman N Antimicrobial stewardship Am J Med 2006 16 S53 S61 10.1016/j.amjmed.2006.04.003 16735152

[B18] Burke JP Antibiotic resistance: squeezing the balloon? JAMA 1998 16 1270 1271 10.1001/jama.280.14.1270 9786379

[B19] McGowan JE Jr Finland M Usage of antibiotics in a general hospital: effect of requiring justification J Infect Dis 1974 16 165 168 10.1093/infdis/130.2.165 4842338

[B20] Raineri E Pan A Mondello P Acquarolo A Candiani A Crema L Role of the infectious diseases specialist consultant on the appropriateness of antimicrobial therapy prescription in an intensive care unit Am J Infect Control 2008 16 283 290 10.1016/j.ajic.2007.06.009 18455049

[B21] Bornard L Dellamonica J Hyvernat H Girard-Pipau F Molinari N Sotto A Roger PM Bernardin G Pulcini C Impact of an assisted reassessment of antibiotic therapies on the quality of prescriptions in an intensive care unit Med Mal Infect 2011 16 480 485 10.1016/j.medmal.2010.12.022 21778026

[B22] Patel SJ Saiman L Duchon JM Evans D Ferng YH Larson E Development of an antimicrobial stewardship intervention using a model of actionable feedback Interdiscip Perspect Infect Dis 2012 16 150367 22500166 10.1155/2012/150367 PMC3303556

[B23] De Angelis G Restuccia G Cauda R Tacconelli E How could we reduce antibiotic use in critically ill patients? Infect Disord Drug Targets 2011 16 376 383 21679144 10.2174/187152611796504791

